# Development and implementation of an MRI‐only simulation, planning, and treatment workflow for prostate radiotherapy using synthetic CT on MR‐linac

**DOI:** 10.1002/acm2.70499

**Published:** 2026-02-09

**Authors:** Reza Reiazi, Yao Ding, Sarath Vijayan, Jinzhong Yang, Ergys Subashi, Yao Zhao, Belinda M. Lee, Hunter L. Emory, Vi T. Dinh, Greg L. Swiedom, Jie Deng, Mu‐Han Lin, Peter Balter, Rajat J. Kudchadker, Elaine E. Cha, Seungtaek Choi, Yusung Kim, Eun Young Han, Surendra Prajapati

**Affiliations:** ^1^ Department of Radiation Physics The University of Texas MD Anderson Cancer Center Houston Texas USA; ^2^ Department of Radiation Oncology Therapy The University of Texas MD Anderson Cancer Center Houston Texas USA; ^3^ Department of Radiation Oncology The University of Texas Southwestern Medical Center Dallas Texas USA; ^4^ Department of Radiation Oncology The University of Texas MD Anderson Cancer Center Houston Texas USA; ^5^ Department of Genitourinary Radiation Oncology The University of Texas MD Anderson Cancer Center Houston Texas USA

**Keywords:** MR‐only simulation, prostate, radiotherapy, synthetic CT

## Abstract

**Purpose:**

We evaluated the feasibility of a magnetic resonance (MR)‐only simulation, planning, and treatment (MROSPT) workflow for prostate cancer patients using synthetic computed tomography (sCT) generated from magnetic resonance imaging (MRI) data. By validating sCT‐based dose calculations, we aimed to streamline radiotherapy workflows, eliminate the need for CT simulation, and enable reliable clinical implementation of MR‐based radiotherapy for MR‐linac (MRL).

**Methods:**

We developed a comprehensive workflow encompassing the entire process from initial consultation to treatment delivery. After developing the workflow, a retrospective dosimetric validation study was performed on nine men with prostate cancer. They underwent CT and MRI simulations, and sCTs were generated from the MRI data. Contours and intensity‐modulated radiation therapy treatment plans were created on the reference simulation CT (rCT) and transferred to sCTs for dose‐calculation comparisons. Dosimetric accuracy was evaluated using gamma analysis (dose/distance; 2%/2mm). Bulk density sCTs (bCTs) were created by overriding organ density values with their mean (bulk) sCT‐determined densities. bCT based on sCT allows treatment planning directly on MRI for MRL workflow efficiency.

**Results:**

Minimal non‐bone Hounsfield units (HU)‐value differences between rCT and sCT (5.5 ± 2.9 HU for prostate) demonstrated the reliability of the sCT generation process. Dosimetric comparisons between treatment plans (rCT vs. sCT, rCT vs. bCT) showed agreement within ± 2% in gamma analysis, confirming robust accuracy. The gamma index pass rate for rCT versus sCT and rCT versus bCT were consistently > 95% using 2%/2 mm criteria. A dry run of the entire simulation‐to‐treatment workflow was successfully completed.

**Conclusion:**

The MROSPT workflow using sCT is clinically feasible and dosimetrically accurate for prostate cancer patients. Dose calculations based on sCT demonstrated high dosimetric agreement with simulation CT, with no statistically significant differences across all evaluated metrics. These findings support the adoption of sCT‑based planning for prostate cancer radiotherapy and suggest its potential applicability in other anatomical regions especially in the pelvis. Integration of robust quality‑assurance processes and treatment‑delivery flexibility will further enhance its clinical utility.

## 1 INTRODUCTION

Magnetic resonance imaging (MRI), an essential tool in radiation therapy, offers unparalleled soft‐tissue contrast compared with computed tomography (CT). This advantage is particularly noteworthy in the treatment of pelvic cancers, for which accurate delineation of target volumes and organs at risk (OARs) is critical.

The integration of magnetic resonance linear accelerators led to the development of the MR‑linacs (MRL), enabling high‑resolution MRI to be merged with real‑time treatment delivery.^1^, ^2^ MRL enables adaptive radiotherapy, allowing clinicians to account for anatomical changes during treatment.^3^, ^4^ In addition, this real‐time, imaging‐guided approach substantially enhances the precision of radiation‐dose delivery, particularly for mobile targets or targets near critical structures.^5^, ^6^

Both traditional and MRL workflows typically rely on both MRI and CT simulation scans, with CT providing essential electron density (ED) information for calculations of radiation doses. Although this dual‐modality approach is effective, it introduces several challenges, including the need for accurate MRI‐CT registration, potential spatial mismatches, workflow inefficiencies, and increased radiation exposure for patients.

Researchers have addressed these issues by using synthetic CT (sCT) technology to generate electron‐density maps directly from MRI data, eliminating the need for CT imaging.^3^, ^4^ Advances in machine learning and imaging have improved the geometric fidelity and dosimetric accuracy of sCT, making an MRI‐only workflow a viable option.^5^, ^6^, ^7^, ^8^, ^9^, ^10^, ^11^, ^12^, ^13^, ^14^, ^15^, ^16^

This study evaluated the feasibility of implementing an MRI‐only simulation, planning, and treatment (MROSPT) workflow for patients with prostate cancer treated on an MRL unit. We also aimed to validate the dosimetric accuracy of sCT‐based dose calculations compared with rCT‐based planning. We sought to highlight the potential clinical and operational benefits of using MRI‐only radiotherapy. The development of this innovative, streamlined workflow represents a significant step towards integrating advanced imaging technologies, such as MRL, into routine clinical practice.

## 2 METHODS

### 2.1 Proposed workflow

We proposed an MRI‐only simulation and treatment (MROSPT) workflow incorporating sCT technology for accurate dose calculations generated directly from MRI data. The workflow consisted of the following steps:

#### 2.1.1 Consultation and preparation

Prior to simulation, consultation sessions were conducted to assess patient eligibility for the MROSPT workflow, excluding individuals with:

- Medical implants or conditions that contraindicated safe MRI scans.
- Body sizes exceeding the bore and coil dimensions of the MRL clearance jig (perimeter > 49 inches at the iliac crest).
- Significant metal artifacts, such as those caused by hip prostheses or other pelvic implants, which could interfere with imaging quality.
- Excessive gas in the bowel or rectum, which could compromise MRI accuracy.

A simulation order was then placed for eligible patients, who were provided with an MRI‑screening form and detailed preparation instructions—including bladder‑filling protocols—to ensure reproducible anatomical conditions. The eligibility assessment involved asking patients to provide informed consent for MROSPT, acknowledging the unique aspects of the workflow. Those who met the criteria were transitioned to preparation protocols specifically tailored to MROSPT workflow, while those excluded were prepared following standard CT‐simulation protocols to maintain consistency and ensure accurate treatment planning.

To further optimize patient selection and workflow reliability, we expanded our screening and preparation protocols. In addition to excluding patients with contraindicated implants, excessive body size, or significant metal artifacts, we also considered borderline cases such as prior pelvic surgeries, stents, or difficulty complying with bladder and rectal preparation. Patients with claustrophobia or limited tolerance for MRI duration were evaluated individually, and alternative workflows were recommended when necessary.

Preparation protocols were reinforced through patient education and coaching. For bladder filling, patients were instructed to void one hour before simulation and drink 16–20 oz of water, with bladder volume confirmed via pre‐scan imaging. Rectal emptying was supported by dietary guidance (low‐residue diet 24–48 h prior) and optional use of mild laxatives or enemas. These steps were critical to ensure reproducible anatomy and minimize gas‐related artifacts, which can compromise MRI quality and sCT generation.

#### 2.1.2 MRI‐only simulation

**FIGURE 1:**
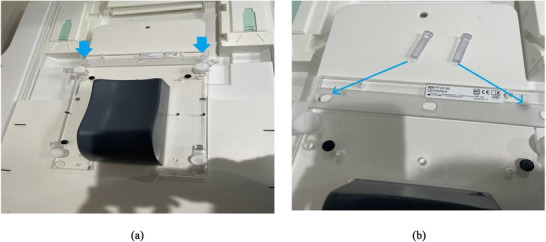
MR sim custom head rest jig with blue arrows pointing to the location of pegs connecting the jig to the MRI table with a full view of the jig (a) and zoomed in view (b) of the jig showing the custom pegs.

**FIGURE 2:**
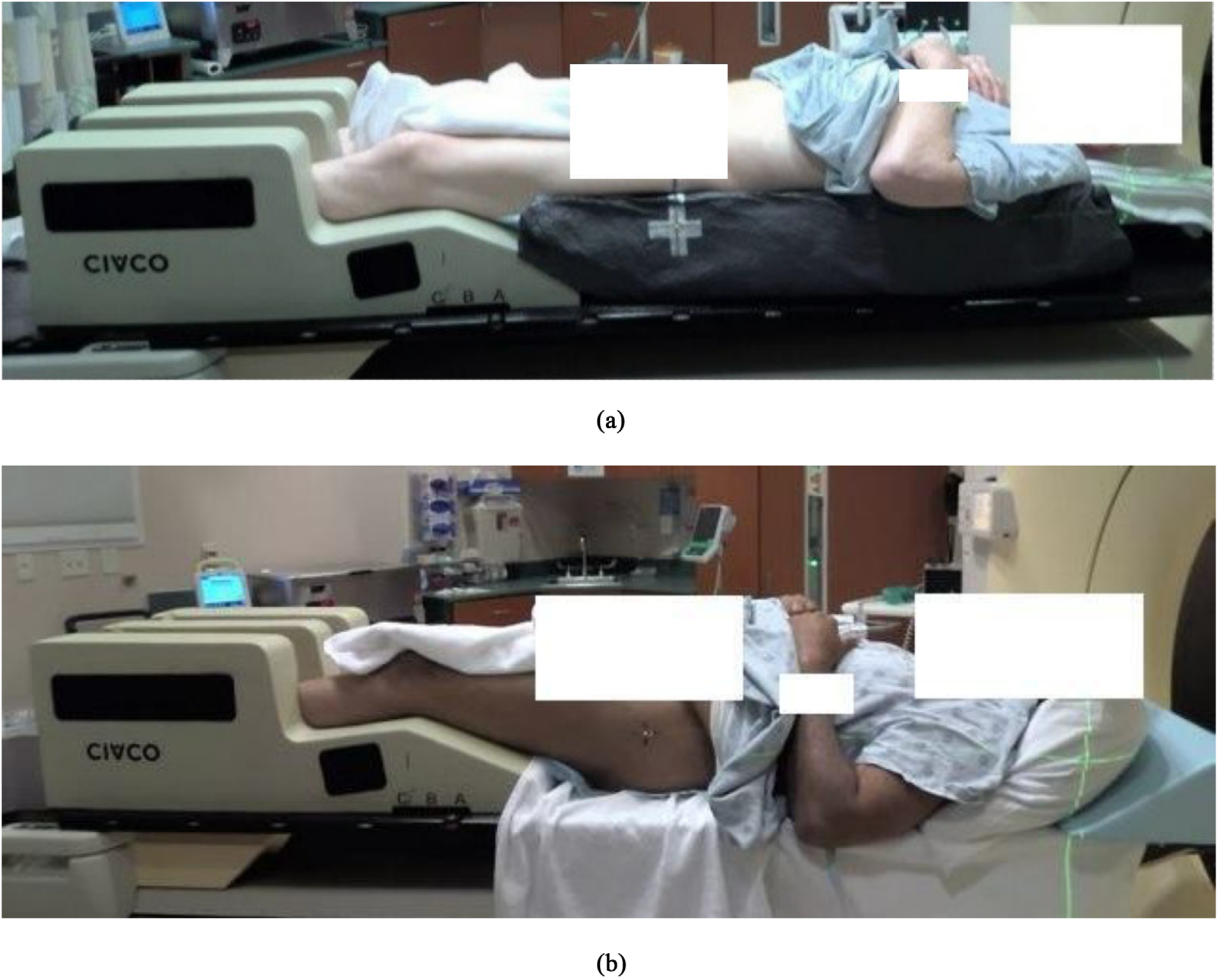
Patient set‐up for simulation for treatment with (a) and without (b) vacloc.

To ensure precise indexing for MRI‐only simulation patients, a custom headrest jig with pins was fabricated in collaboration with our institution's machine shop. When we “zero” the MRI sim longitudinal axis at the indexing position of the jig, we can calculate the longitudinal position of the marked iso with respect to the jig position, which can be converted to the MRL couch index location. Hence, this custom jig (Figure 1) enables accurate couch indexing for seamless transfer to the MRL system (Unity, Elekta AB, Stockholm, Sweden) during treatment. Patients were positioned with their heads resting on standard headrests, hands on their chests, bodies supported by vacloc immobilization bags (optional), and legs on a Medtec leg‐immobilization device (Figure 2). Then bladder scans were performed to confirm appropriate bladder volume before simulation. A 3 Tesla Vida MRI scanner (Siemens Magnetom Siemens Healthineers, Erlangen, Germany) was designated as the MRI‐only simulation scanner. It is equipped with external lasers and features a flat couch top similar to that of a CT‐simulation scanner, ensuring compatibility with standard radiotherapy workflows.

Since vacuum pumps cannot be housed in Zone 4 of an MRI suite due to safety restrictions, a customized, 110V vacuum pump with an Airswitch handheld control switch, featuring 2 channels and a 30‐ft hose (Klarity KVP‐500 MDA), was developed in collaboration with Klarity Medical Products, LLC (Newark, Ohio). The pump is tethered to the floor in the Zone 3 console room, and the long hose and air switch allow therapists to control the non‐MRI‐safe vacuum pump safely within the MRI suite during patient setup. The generation of sCT images required specific MRI sequences, particularly the T1‐weighted VIBE DIXON protocol,^17^ which was optimized to provide both high soft‐tissue contrast and quantitative information correlating with ED. Because the MRL has no lasers to align the marked isocenter for treatment setup, a lookup table (LUT) was developed for positioning the patients at MRI simulation so that the marked isocenter could be correlated with indexed couch parameters for treatment on the MRL. We followed the same procedure to develop LUT for MRL couch indexing reported by Hughes et al.^18^ For imaging, 18‐channel flex coils were used to enhance image quality, and setup images were captured from outside the 5‐Gauss line to ensure patient safety.

#### 2.1.3 sCT generation

sCT images were generated from MRI data using Siemens’ syngo.via RT Image Suite software.^18^ The resulting sCT was validated for geometric accuracy and dosimetric reliability to ensure it met clinical standards for use in treatment planning, particularly those using MRI‐only workflows and MRL systems. The software used spectral information to classify tissue types and identify bone structures through a multi‐atlas‐based model and generated sCT datasets, including a continuous electron‐density map for each tissue type.

Once created, the sCT images were exported to the picture‐archiving and communications system (PACS) system, and the patient's table position from the simulation session was transferred to the MRL table using in‐house couch‐indexing guidelines.

#### 2.1.4 Treatment planning and delivery

Treatment planning was conducted using axial, T2‐weighted MRI images, with the mean ED for each organ extracted from the sCT dataset and mapped to the corresponding structures on MRIs. A comprehensive quality‐assurance process validated the transfer of the mean ED values and ensured that the plan met all clinical and safety standards. To ensure clinical safety and dosimetric reliability, the transfer of mean ED values from the sCT to the MRI structures underwent a multistep quality assurance (QA) process. First, the mean ED values for each organ‐at‐risk (OAR) and target structure were extracted from the continuous ED map generated by the sCT. These values were reviewed against expected mean ED value relative to water derived from institutional consensus data including prostate (1.04), bladder (1.01), urethra (1.03), sigmoid (0.98), rectum (1.00), seminal vesicle (1.01), and femoral head (1.2). Any deviations beyond ± 3% will trigger a secondary review and re‐segmentation if necessary.

The rationale for these thresholds was based on internal benchmarking of CT‐derived ED distributions across over 100 pelvic cancer patients, ensuring that the sCT ED values remained within clinically acceptable limits. Additionally, the bulk ED values were verified within the treatment planning system by recalculating dose distributions and comparing them to reference CT‐based plans using gamma analysis. This dual‐layer QA—numerical validation and dosimetric comparison—ensured that the transferred ED values supported safe and accurate dose delivery. The process was integrated into the clinical workflow and reviewed by physics prior to plan approval.

The proposed MROSPT workflow is designed for clinical implementation on the MRL. In this workflow, patients would be planned on MRI images and treated using an MRI‑only reference plan, with the online or offline adaptive process beginning with daily MR imaging to verify patient setup and bladder filling, followed by contour and plan adaptation to account for anatomical variations and then generate an optimized treatment plan for precise delivery. For this study, however, the nine patients used for dosimetric validation were treated clinically at MRL using the conventional CT‑based reference plan. The corresponding MRI‑only plans were generated retrospectively and evaluated offline to assess the feasibility and dosimetric accuracy of the proposed MROSPT workflow.

### 2.2 Dosimetric validation of dose calculations on sCTs data using Siemens’ syngo.via RT image suite software

To validate the dosimetric accuracy of the MROSPT workflow, a dosimetric comparison study was done for the nine men with prostate cancer. These patients underwent both CT and MRI simulations, enabling a comparison of dosimetric accuracy between CT‐based and MRI‐only workflows. The study was approved by the institutional review board under protocol number PA14‐1002.

For treatment planning, rCT scans and T2‐weighted MRI images were imported into the treatment‐planning system (TPS) (Monaco, version 6.2.2.0, Elekta AB, Stockholm, Sweden), and contouring was performed on the CT images using rigid registration with MRI images. The sCT images were then deformably registered to the CT simulation dataset using MIM software (MIM Software Inc, Cleveland, Ohio) to ensure consistent geometry for a direct dose comparison between the MRI and CT simulation datasets. Following registration, the contours from the CT dataset were transferred to the sCT to ensure accurate alignment of the target and OAR structures across both datasets.

The treatment‐planning process involved the development of 3 different treatment plans generated from different sets of images: 1) an rCT treatment plan, based on the electron densities from rCT scans; 2) an sCT treatment plan, generated from the continuous electron‐density map derived from sCT images and for which the rCT plan was copied, applied to the sCT dataset, and recalculated without any change or re‐optimization; and 3) a bCT treatment plan, which was derived from the mean (bulk) density values for all regions of interest (ROIs), calculated from the continuous electron‐density map from the sCT, and allowed treatment planning directly on MRI for an efficient MRL workflow. Like the sCT treatment plan, the rCT treatment plan was copied and applied to the bCT dataset, and recalculated without any changes or re‐optimization.

The remaining steps of the planning process followed standard MRL workflows. To ensure consistency and allow unbiased comparisons, we used identical treatment parameters—including control points—across all plans. We also confirmed that the iso‐coordinates of the treatment isocenters were identical. Upon dose calculation, all plans were systematically reviewed and compared within the TPS for dose‐volume histogram (DVH)‐related dose statistics. Additionally, a 3‐dimensional gamma index analysis was performed to quantitatively assess the agreement between the sCT‐based plans and the rCT‐based plans.

We also completed a successful dry run of the entire MROSPT workflow for patients with prostate cancer, focusing on intact‐prostate treatment plans and evaluating dose agreements for the entire pelvic region.

## 3 RESULTS

Implementation of the MROSPT workflow successfully streamlined the clinical process for MRL‐based treatment of prostate cancers, eliminating the need for CT simulation and enabling MRI‐only planning and delivery. Compared to the conventional CT‐based workflow, the MROSPT workflow introduced key modifications at each stage of the treatment pathway, which were successfully integrated into clinical practice. The main differences include:

1. **Consultation and pre‐simulation**
  ∘ The electronic order process was adjusted to specify *MR‐only simulation* for MRL patients, removing the CT simulation order.
  ∘ MRI evaluation needed to be ordered before simulation to ensure appropriate patient selection and workflow adaptation.
2. **Simulation**
  ∘ Patients followed modified preparation protocols, including rectal emptying and bladder filling checks before the MRI scan.
  ∘ The simulation setup included headfirst supine positioning with modified immobilization strategies, incorporating indexing‐specific devices for the MRL couch.
  ∘ Vacuum bags were not required for immobilization due to MRL constraints, and when used, MR fiducial markers were placed on couch for accurate couch positioning.
  ∘ sCT images were generated from MRI sequences to serve as a substitute for ED maps required for dose calculation.
3. **Planning**
  ∘ Contouring was performed on MRI images rather than CT, with structures mapped to SynCT for dose calculation.
  ∘ The planning workflow incorporated SynCT within the TPS to enable accurate dose calculation and plan adaptation.
  ∘ Two planning strategies were introduced: (1) Using sCT as the primary reference image and (2) using MRI as the reference image while manually assigning bulk electron densities.
4. **Treatment**
  ∘ The treatment workflow incorporated MR‐based online adaptation, utilizing both Adapt‐to‐Position (ATP) and Adapt‐to‐Shape (ATS) approaches to guide patient setup and plan re‐optimization. In the ATP workflow, the reference plan is adjusted based on daily patient positioning using rigid registration, without modifying the anatomical contours. In contrast, the ATS workflow involves deformable image registration and contour propagation to account for daily anatomical changes, enabling re‐optimization of the treatment plan based on the updated anatomy.
  ∘ Patient positioning relied on MRI‐based images.

**FIGURE 3:**
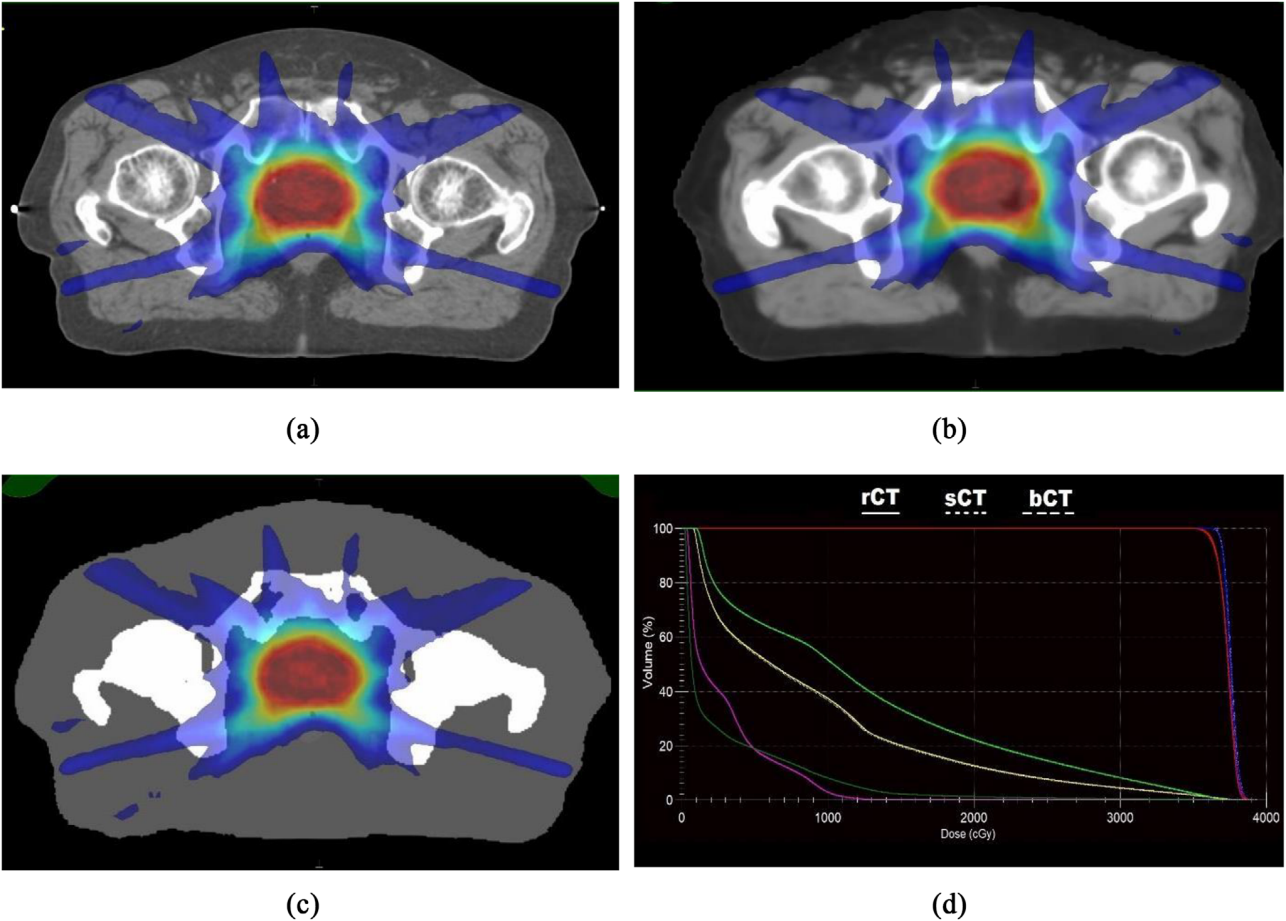
Axial dose distributions of a single treatment plan calculated using (a) reference simulation CT (rCT), (b) sCT, and (c) bCT. DVHs are shown in panel D for the planning target volume(PTV) (red), prostate (blue), rectum (light green), bladder (yellow), right femur (magenta), and total body (dark green). All DVHs are mostly overlapping with negligible dose differences.

For dosimetric comparison, rCT and the two sCT‐based planning methods—sCT and bCT—were evaluated. Figure 3 illustrates the axial dose distribution of a single treatment plan using rCT, sCT, and bCT and DVH comparison.

**FIGURE 4:**
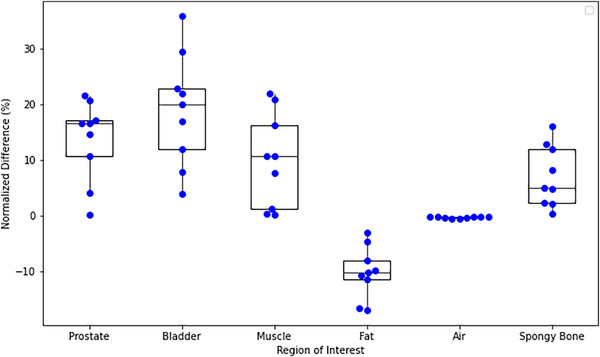
Swarm plot overlaid with a box plot showing the normalized differences in HUs between the reference and simulation CT and sCT for various ROIs.

Minimal non‐bone differences in HU values between rCT and sCT (5.5 ± 2.9 HU for the prostate, *p*‐value > 0.01) demonstrated the reliability of the sCT generation process. The average differences in HU values across ROIs were as follows: prostate (5.5), bladder (3.8), muscle (5.0), fat (10.1), spongy bone (75.1), and air (2.0). These differences are shown in Figure 4.

**FIGURE 5:**
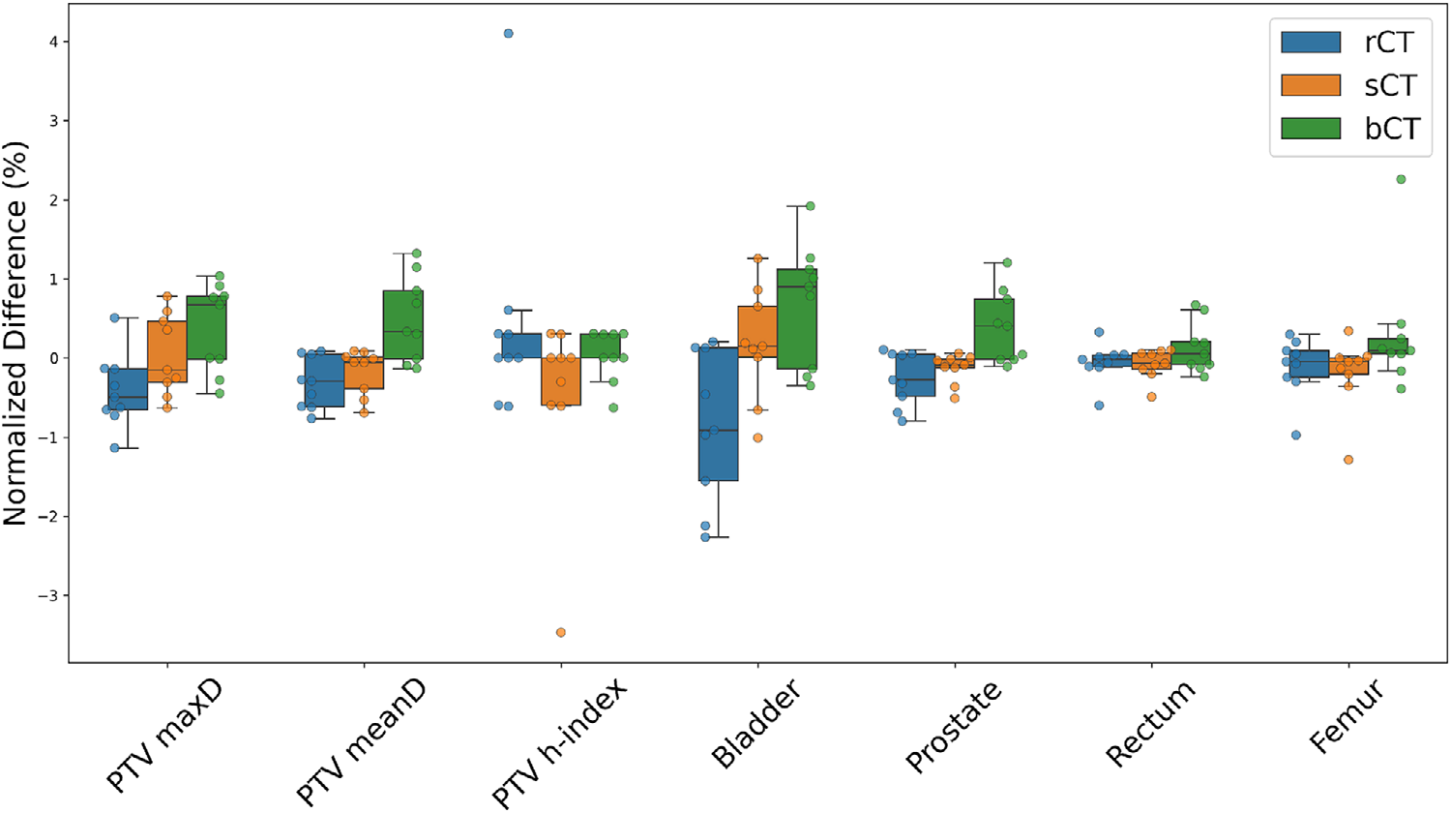
Summary of PTV dose parameters (maxD, meanD, and homogeneity index [h‐index]) across different scenarios and meanD values for critical structures. All values were normalized to the mean value across the different plans. There were no statistically significant differences between the different plans. bCT; rCT; sCT.

The results also revealed key variations in dose metrics across the treatment plans using the rCT, sCT, and bCT datasets. These variations were observed across multiple structures and dose‑volume parameters. Figure 5 provides a summary of the calculated doses to the target (PTV mean dose (meanD), PTV maximum dose (maxD), PTV heterogeneity index, and prostate) and critical structures (rectum, bladder, and femur) and highlighted the dosimetric impact on the target and the OARs. No significant differences were observed between the sCT and bCT plans when compared with the rCT plans, confirming the dosimetric accuracy of sCT‐based planning. Pairwise comparisons of the dose parameters across the treatment plans (rCT, sCT, and bCT) were performed using the Wilcoxon signed‐rank test, as the data did not meet normality assumptions. No statistically significant differences were found between the plans; *p*‐values were greater than 0.01 for all comparisons.

**FIGURE 6:**
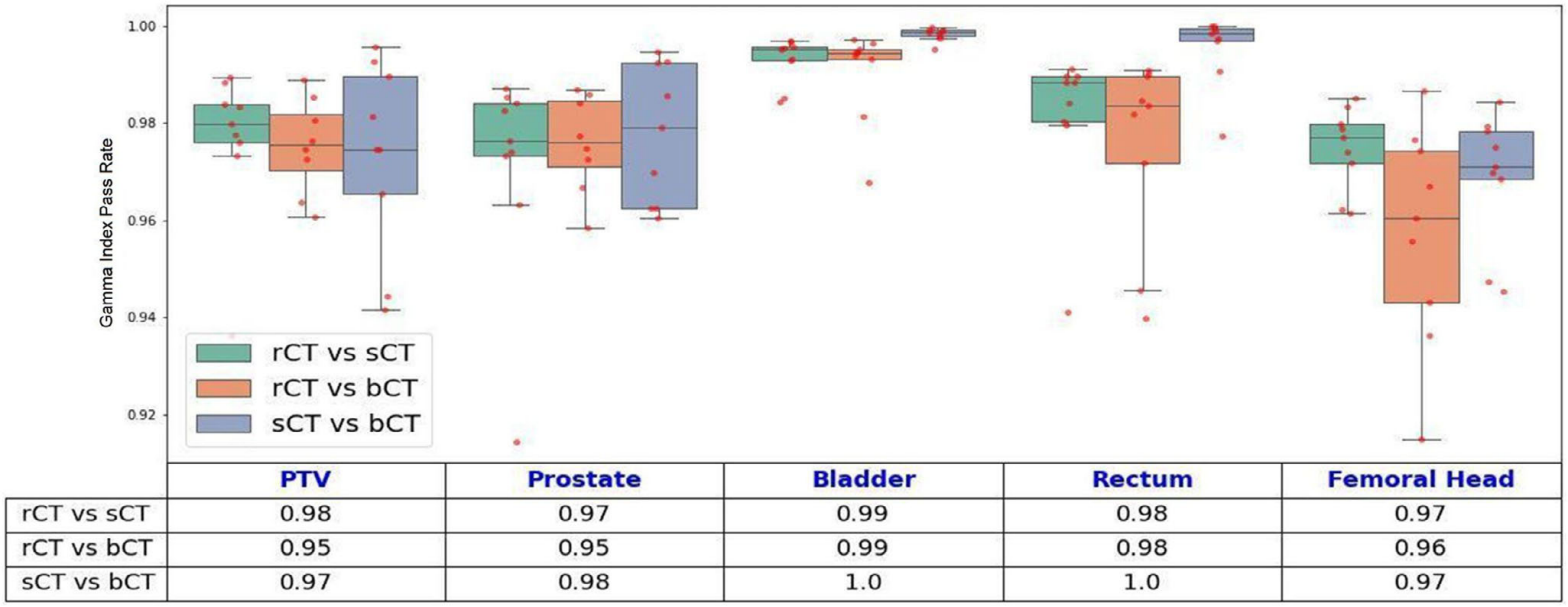
Comparison of the gamma index pass rate for key ROIs, including the bladder, rectum, prostate, femoral heads, and PTV, across different imaging‐based plans. Boxplots illustrate the distribution of gamma index pass rates for each ROI, with red dots indicating individual patient data points. The accompanying table summarizes the gamma index pass rates for each anatomical mask and imaging approach, emphasizing dose calculation differences between methods. Institutional action level for gamma analysis is set at 95% pass rate (2%/2mm criteria, global normalization, dose threshold 10%), below which the plan is considered to fail. bCT, rCT, sCT.

**FIGURE 7:**
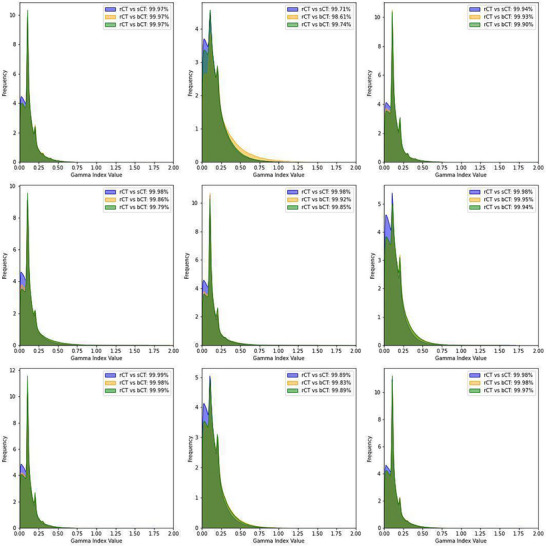
Comparison of the gamma index pass rate distributions for total body across multiple cases for CT: rCT, sCT with continuous mapping, and sCT with bulk density (bCT) assignment. Each subplot corresponds to an individual patient case, with the histogram of the gamma index pass rate, representing frequency distributions of voxel‐wise comparisons. The blue histograms indicate the comparison between rCT versus sCT, the orange histograms represent rCT versus bCT, and the green histograms show sCT versus bCT. Reported gamma index pass rates (global 2%/2mm criteria, 10% threshold) demonstrate high agreement across all comparisons, exceeding 98% in all cases.

Figure 6 illustrates the gamma index pass rate ^19^ for the comparisons of the treatment plans. We used a distance/dose 2 mm/2% criterion with a 10% low‐dose threshold for the PTV, bladder, rectum, prostate, and femoral head. The breakdown of the gamma index pass rate for dose calculated for total body for all patients is shown as in Figure 7. The gamma index pass rate for rCT versus sCT and rCT versus bCT were consistently over 95%.

## 4 DISCUSSION

This study focused on prostate cancer for dosimetric validation; however, the MR‐only simulation workflow is broadly applicable to all pelvic malignancies, given the consistent imaging protocols, segmentation strategies, and dose calculation methods across pelvic treatment sites. We developed and dosimetrically validated the MROSPT workflow for prostate cancer MRL treatment, demonstrating its feasibility for clinical implementation. As part of this work, we evaluated commercial software for sCT generation and validated dose calculations using both sCT and bCT to support MR‐based treatment planning. Following this evaluation, we successfully completed a dry run that included MR‐only simulation, treatment planning, and delivery.

Transitioning to an MROSPT workflow required substantial modifications across all stages of the radiation treatment process. The elimination of CT simulation removed direct ED information, necessitating the use of sCT for dose calculation. However, this approach enabled a fully MRI‐based workflow, improving soft tissue contrast and alignment accuracy.

Furthermore, refinements in MROSPT workflow—including optimized immobilization strategies and adjusted simulation protocols—ensured compatibility with the MRL environment. Early validation through hybrid MRI + CT workflows confirmed the feasibility of this approach. Future efforts will focus on extending the workflow to non‐MRL patients and incorporating AI‐driven auto‐contouring tools to enhance efficiency in planning and segmentation.^20^

In the dosimetric validation, the minimal differences observed in the HU values for the treatment plans (except for bone) underscore the reliability of the software in generating sCTs, particularly for soft tissues. However, the large variations for bone indicate that sCTs may require further refinement for high‐density regions. The higher absolute differences between the rCT and sCT HU values for bone can be attributed to several key factors. Bone has a much higher HU range (approximately 700–2000 HU) compared to soft tissues like muscle (approximately 100 HU). Because the absolute HU values for bone are large, even small percentage errors in sCTs result in larger absolute differences. Additionally, MRI signal intensity ^21^ does not correlate well with HU values for high‐density structures like bone, and partial volume effects (where a voxel contains multiple tissue types) can lead to greater errors in sCT estimation.^20^, ^22^ Incorporating soft objectives to limit beam entrance paths through the femoral heads can help mitigate HU uncertainties in dose calculations, reducing potential inaccuracies associated with intensity‐modulated radiation therapy beams passing through bony structures.

Our analysis showed no significant differences in dose distributions across the evaluated plans. Generally, the bCT plans exhibited slightly higher maximum and meanDs for the PTV, suggesting a tendency for dose overestimation with bCT. The heterogeneity index remained stable across all plans, although slight variations in dose uniformity were observed within the PTV. For OARs such as the bladder, prostate, rectum, and femur, the bCT plans showed marginally higher doses than the sCT and rCT plans, likely due to differences in tissue representation and dose‐calculation accuracy. The sCT plans closely mirrored the rCT plans, indicating strong alignment in dose delivery; small deviations between the plans, particularly in high‐density regions like bone and small structures, likely arose from errors introduced by the deformable image registrations used for dosimetric comparison and from interpolation issues from the resampling of coarse dose grids. These discrepancies are more pronounced in small structures due to the high dose gradient, particularly around the edges of the PTV, where small misalignment can significantly affect the dose and gamma index pass rate.

Our findings align with those of previous studies demonstrating the feasibility and advantages of sCT for MRI‐only radiotherapy workflows.^7^, ^8^, ^9^, ^10^, ^11^, ^12^, ^13^, ^14^, ^15^, ^16^ sCT streamlines treatment planning, reduces radiation exposure, and mitigates registration errors, supporting its clinical adoption for various anatomical sites, including the prostate and the pelvis. High gamma index pass rates and minimal differences in dose statistics across different dose calculation scenarios (rCT vs. sCT) reinforce the dosimetric reliability of sCT.

Our study provides additional insight by including bCT as a third planning approach. The inclusion of bCT is particularly relevant because bulk‐density assignment is one of the commercially supported options for MRI‐only workflows on the Unity MRL platform. By incorporating bCT into our analysis, we aimed to evaluate a simplified yet clinically applicable method of electron‐density assignment, which may further streamline the MR‐only planning process. This approach allows treatment planning directly on MRI data without the need for a detailed continuous electron‐density map, potentially reducing computational demands and simplifying clinical implementation. Our data demonstrated that both the sCT and bCT workflows yielded dosimetric results comparable to those of conventional CT‐based planning, reinforcing the clinical feasibility of these MRI‐only strategies and providing evidence to support bCT as a viable alternative in MRI‐only workflows. These findings extend the current literature and support the adoption of either method depending on institutional preferences and workflow considerations.

Although this study demonstrated the feasibility and dosimetric accuracy of the proposed MROSPT workflow for patients with prostate cancer, certain limitations should be noted. The small sample size of nine patients, while adequate for dosimetric comparisons, limits the generalizability of the findings. In addition, this work reflects a single‑institution experience using a single‑vendor MRI unit and MRL platform (Siemens/Unity). Broader multi‑institutional data would be needed to further strengthen external validity. Deformable image registration, used for alignment of rCT, sCT, and bCT, introduced potential errors, particularly in high‑density regions like bone and in small ROIs in high‑dose‑gradient areas. However, as deformable image registration is not part of the clinical MROSPT workflow, these uncertainties do not impact the workflow's practical implementation. Slightly lower gamma index pass rates for the PTV and structures such as the femoral head further highlight the potential impact of interpolation errors and dose‑grid‑resolution differences.

Despite these limitations, our findings support the reliability of the sCT‐based workflow in eliminating CT‐based simulation, reducing registration‐related uncertainties, and improving the efficiency of radiotherapy planning and treatment delivery. Extending this workflow to other anatomical sites will require additional validation. With these milestones achieved, the workflow is now prepared for clinical implementation. Future developments will focus on extending this workflow to non‐MRL patients and incorporating auto‐contouring in MRI to further streamline the process.

## 5 CONCLUSION

This study supports the dosimetric accuracy and feasibility of the MRI‐only radiotherapy workflow using sCT for prostate cancer treatment. The minimal differences observed between treatment plans in terms of the HU values (except in bone) and dose distributions support the reliability of sCT in soft‐tissue representation while highlighting the need for refinement in high‐density regions. The successful implementation of this workflow demonstrates its potential to streamline treatment planning, reduce radiation exposure, and enhance patient comfort. Further validation across diverse anatomical sites is required to fully establish its clinical applicability and expand its use in radiotherapy.

## AUTHOR CONTRIBUTIONS

**Reza Reiazi**: Conceptualization, Methodology, Formal analysis, Investigation, Data curation, Visualization, Writing—original draft. **Yao Ding**: Methodology, Software, Formal analysis, Writing—review and editing. **Sarath Vijayan**: Writing—review and editing. **Jinzhong Yang**: Resources, Validation, Writing—review and editing. **Ergys Subashi**: Resources, Writing—review and editing. **Yao Zhao**: Data curation, Investigation, Writing—review and editing. **Belinda M Lee**: Data curation, Writing—review and editing. **Peter Balter**: Resources, Writing—review and editing. **Rajat Kudchadker**: Writing—review and editing. **Elaine E Cha**: Investigation, Writing—review and editing. **Seungtaek Choi**: Conceptualization, Resources, Writing—review and editing. **Yusung Kim**: Conceptualization, Supervision, Writing—review and editing. **Eun Young Han**: Conceptualization, Supervision, Project administration, Writing—review and editing. **Surendra Prajapati**: Conceptualization, Methodology, Supervision, Project administration, Writing—review and editing.

## CONFLICT OF INTEREST STATEMENT

The authors declare no conflicts of interest
